# Genetic and Environmental Effects on Parent‐Rated Adaptive Behaviour in Infancy

**DOI:** 10.1111/desc.70041

**Published:** 2025-06-19

**Authors:** Hanna Halkola, Charlotte Viktorsson, Emily J. H. Jones, Tony Charman, Terje Falck‐Ytter, Giorgia Bussu

**Affiliations:** ^1^ Department of Psychology, Institute of Psychiatry, Psychology and Neuroscience King's College London London UK; ^2^ Department of Psychology, Development and Neurodiversity Lab Uppsala University Uppsala Sweden; ^3^ Department of Psychological Sciences, Centre for Brain and Cognitive Development Birkbeck, University of London London UK; ^4^ Department of Women's and Children's Health, Karolinska Institutet & Stockholm Health Care Services Center of Neurodevelopmental Disorders (KIND), Centre for Psychiatry Research Stockholm Sweden; ^5^ Swedish Collegium for Advanced Study Uppsala Sweden

**Keywords:** adaptive behaviour, aetiological structure, development, infancy, multivariate, twins

## Abstract

Adaptive behaviour refers to the everyday skills that individuals are expected to have to function independently, based on their age and societal norms. Currently, we know little about the role of genetic and environmental factors in parent‐rated adaptive behaviours in early infancy. The aim of this study was to investigate the aetiological factors that influence individual variability in different adaptive behaviour domains at 5 months, and the degree of genetic and environmental influences that are unique and shared across these domains. We analysed data from the Vineland Adaptive Behaviour Scale (VABS‐II) motor domain and combined domain of socialization and communication (social‐communication) using a multivariate twin modelling approach. Participants were a community sample of monozygotic and dizygotic twins assessed at 5 months of age (*n* = 594). The results show high shared environmental influence on both motor (0.67) and social‐communication (0.78) domains with 45% shared variance. Both had low, but significant heritability estimates (0.21 and 0.12, respectively) but did not share genetic variance. No statistically significant associations were found between polygenic scores for autism, ADHD, schizophrenia, depression, and bipolar disorder, and either of the adaptive behaviours measured here. Our results highlight the importance of shared environmental factors in the development of social‐communication and motor skills in infancy, whether it is through social interaction with caregivers, or the stimuli and opportunities presented at home.

**Summary:**

- During development structural arm length representation is underestimated, while the functional arm length representation is overestimated.
- Underestimation of structural arm length is driven by an underestimation of hand length, as forearm length is accurate.
- Structural hand length is underestimated, supporting that underestimation of hand length is a characteristic of human body representation.
- The opposite pattern of results between structural and functional arm representation suggests the existence of multiple independent representations of the body.

## 1 Introduction

Adaptive behaviour is the performance level of daily activities required for personal and social sufficiency that is expected of the individual's age (Schalock et al. 2010). It describes the typical behaviour in everyday life, not the maximum capabilities of the individual. These skills are building blocks in forging and maintaining relationships, selfcare and completing everyday tasks. Adaptive behaviour can be split into four domains: (1) communication, comprised of expressive, receptive, and written language skills; (2) socialization, which includes interpersonal relationships, play and leisure time, and coping skills; (3) daily living skills regarding personal, domestic and community context; and (4) motor skills, which contain both fine and gross motor abilities (Sparrow et al. 2005). Although adaptive behaviour is most often studied in older children and adults, it can be assessed already in infancy. Adaptive behaviour in infancy includes behaviours such as head support, crawling, turning towards sounds, responding to caregiver, and expressing emotions and needs through crying, smiling and other sounds or gestures.

Twin modelling and polygenic scores offer an opportunity to investigate the heritability of adaptive behaviour, in addition to environmental factors. Understanding these influences on adaptive behaviour would not only deepen our understanding of the aetiology but also help in supporting the development of these important skills starting from infancy. Previous studies in different domains of adaptive behaviour and cognitive development have found that domains related to communication and socialization have low to moderate heritability in early childhood (Austerberry et al. 2022). In a meta‐analysis of developmental milestones from birth to the age of 2 by Austerberry et al. (2022), pooled heritability estimates for basic interpersonal functions and language were 0.38 and 0.24, respectively. However, these estimates might increase over time, as Cheung et al. (2015) found that the heritability of communication and socialization increases throughout the first 6 years of life from low heritability to high, with the shared environment explaining most of the individual variability in early childhood and decreasing towards mid‐childhood. On the other hand, Austerberry et al. (2022) found that basic interpersonal function had a pooled shared environmental estimate of 0.21, while language had an estimate of 0.59. Meanwhile, the pooled unique environmental estimates were 0.41 and 0.18, respectively. Furthermore, Dale et al. (2000) found lexical and grammatical knowledge has been found moderately heritable at 2 years of age, while shared environment explained around 69% of the variance. However, the heritability estimates increase when looking at toddlers with language delays (Dale et al. 1998). Meanwhile, Bussu et al. (2023) found that only 10% of the variance of language skills was explained by shared environment and 25% by genetic influences in 5‐month‐old infants.

Compared to social and communication skills, heritability of motor skills seems to be more constant across infancy (Austerberry et al. 2022). Austerberry et al. (2022) found that psychomotor skills were largely explained by genetic influence (pooled estimate of 0.59), with remaining variance mainly explained by unique environment (0.33), and a small influence of shared environment (pooled estimate of 0.07). Cheung et al. (2015) found that the heritability of gross motor development during the first 6 years of life kept stable with high estimates with little shared environmental influence. For fine motor development, both genetic and shared environmental effects were significant, with the genetic influence increasing and shared environmental influences decreasing over time (Cheung et al. 2015).

While heritability estimates provide information about the genetic influence of variation between individuals in a sample, polygenic scores are a molecular genetics method that utilizes DNA to estimate an individual's inherited likelihood of complex traits and conditions. These scores are comprised of the sum of the common genetic variation linked to specific traits (e.g., neurodevelopmental conditions) through genome‐wide association studies (GWAS's) and aggregated at an individual level across the entire genome (Plomin and von Stumm 2022). Polygenic scores might provide a deeper understanding of genetic contributions to adaptive behaviour, whereas twin models provide insight on the influences of heritability and environment on adaptive behaviour. In early infancy, polygenic scores could indicate if atypicalities in early adaptive behaviour could be an early marker for later neurodevelopmental or psychiatric conditions. Difficulties in adaptive behaviour have been associated with different neurodevelopmental conditions, including autism (Perry et al. 2002), ADHD (Roizen et al. 1994) and intellectual disability (Schalock et al. 2010). For example, infants with a later diagnosis of autism also demonstrated lower adaptive behaviour at 12 months of age compared to their typically developing peers (Estes et al. 2015) and that children with a family history of autism and with a later diagnosis of autism had significantly lower fine motor (Bussu et al. 2018) and gross motor skills (Estes et al. 2015) than their typically developing peers in infancy.

Studies looking at polygenic scores of neurodevelopmental and psychiatric conditions have found associations between social difficulties in childhood and adolescence and polygenic scores for autism, ADHD, major depression, and schizophrenia (Schlag et al. 2022). Polygenic scores for ADHD have been associated with larger infant expressive vocabulary at 15–18 years of age (Verhoef et al. 2024), as well as language difficulties at 5 years of age (Askeland et al. 2022). Meanwhile, autism polygenic scores have been found to associate with language difficulties as early as 18 months (Askeland et al. 2022). Polygenic scores for schizophrenia, in turn, have been found to associate with both social and behaviour difficulties at the age of 4 (Riglin et al. 2017). Regarding motor development, polygenic scores for autism has been found to associate with less‐than‐optimal neuromotor functions at the age of 9–20 weeks (Serdarevic et al. 2020) and motor difficulties at 36 months (Askeland et al. 2022). In turn, the polygenic score for ADHD has been found to associate with earlier walking age (Hannigan et al. 2023). Fine motor skills in early childhood have been found to negatively associate with polygenic scores for ADHD and positively with polygenic scores for anxiety (Bowler et al. 2024).

There is significant overlap between multiple major neurodevelopmental and psychiatric conditions, such as autism, ADHD, schizophrenia, major depressive disorder, and bipolar disorder (Cross‐Disorder Group of the Psychiatric Genomics Consortium and Cross‐Disorder Group of the Psychiatric Genomics Consortium 2019; Zhao and Nyholt 2017), leading to difficulties in drawing diagnostic boundaries between different conditions. Understanding the aetiological factors underlying individual differences in early stages of adaptive behaviour and the degree of genetic independence or inter‐dependence across the different domains can be beneficial in understanding potential difficulties and help to provide support for individuals and families. In this study we aim to examine the aetiological factors contributing to the different domains of emerging adaptive behaviour (e.g., socialization/communication and motor skills) in infants at 5 months of age through parent reports, testing whether they are independent of each other or if they have common genetic influences. As generally moderate phenotypic correlations between the domains have been previously reported across all ages with higher associations at a younger age (Sparrow et al. 2005), we expected to find positive associations between the domains of adaptive behaviour. From previous literature we expected both genetic and shared environmental effects on social and communication skills, while motor skills would be explained by genetic effects and the non‐shared environment.

Additionally, we explored the association between polygenic scores for a wide range of psychiatric conditions (i.e., autism, ADHD, schizophrenia, depression, and bipolar disorder) and early adaptive behaviour. As difficulties in adaptive behaviour have been linked to neurodevelopmental conditions, we hypothesize that higher PGS scores would be associated with lower adaptive skills as early as 5 months of age (see the pre‐registered analysis plan at https://osf.io/fp4q9/).

## 2 Methods

### 2.1 Participants

The sample consisted of infants from the longitudinal Babytwins Study Sweden (BATSS; Falck‐Ytter et al. 2021). BATSS includes 622 same‐sex twins (311 pairs) that were recruited from the national population registry (in the greater Stockholm area in Sweden). From 2016 to 2020, 311 families (29% of the entire population of same‐sex twins born in the area) participated in the multi‐methods assessment at 5 months. Data collection was done at the Centre of Neurodevelopmental Disorders at Karolinska Institutet (KIND) in Stockholm, Sweden. In general, the study sample has a high socioeconomic status, and it includes mainly Swedish‐born families (90% of twin pairs had at least one parent born in Sweden). See Table 1 for sample demographics (in‐depth demographics are reported elsewhere; Falck‐Ytter et al. 2021). Parents gave informed consent to take part. BATSS was approved by the regional ethics board in Stockholm and was conducted in accordance with the Declaration of Helsinki.

**TABLE 1:** Demographics and descriptive statistics data are reported as mean value (standard deviation) across the entire sample and stratified by zygosity (MZ = monozygotic twins; DZ = dizygotic twins).

	Number of twins		Mean (SD)				
	MZ	DZ	MZ	DZ	Overall		
*N* (females %)	330	264	—	—	594		
	46.06%	50.00%			47.65%		
Age (in days)	328	262	167.06 (8.82)	167.58 (8.98)	167.29 (8.88)		
Term age	332	266	257.89 (7.73)	260.87 (7.68)	259.21 (7.84)		
Average parental age	332	266	35.15 (4.72)	35.48 (4.93)	35.30 (4.81)		
Average parental education^*^	332	266	4.27 (0.76)	4.30 (0.74)	4.28 (0.75)		
Family income^**^	328	256	7.42 (2.36)	7.63 (2.50)	7.51 (2.43)		
**VABS‐II**						Skewness	Kurtosis
Motor skills	326	259	10.17 (1.02)	10.40 (1.07)	10.27 (1.05)	−0.18	−0.089
Social‐communication	326	260	34.16 (1.51)	34.29 (1.81)	34.22 (1.65)	0.34	0.24

*Note*: Skewness is reported for the variable distribution across the entire sample.

^*^ Ranking levels: 1 = not known; 2 = primary; 3 = secondary; 4 = tertiary undergraduate; 5 = postgraduate.

^**^ Ranking levels: 1 = not known; 2 = < 20k SEK; 3 = 20–30k SEK; 4 = 30–40k SEK; 5 = 40–50k SEK; 6 = 50–60k SEK; 7 = 60–70k SEK; 8 = 70–80k SEK; 9 = 80–90k SEK; 10 = 90–100k SEK; 11 = > 100k SEK.

General exclusion criteria for the study were opposite‐sex twin pairs, known presence of a genetic syndrome related to autism, uncorrected vision or hearing impairment, diagnosis of epilepsy, very premature birth (prior to Week 34), presence of a developmental or medical condition likely to affect brain development (e.g., cerebral palsy), and infants where none of the biological parents were involved in the infant's care. After recruitment and testing, three infants were excluded because they subsequently were found not to fulfil the above general criteria due to spina bifida (*n* = 1 infant) and seizures at the time of birth (*n* = 2 infants). In addition, for this analysis we excluded infants due to twin‐to‐twin transfusion syndrome (*n* = 12 pairs) and birthweight below 1.5 kg (*n* = 1 twin). A total of 299 twin pairs were included in the analysis, with 295 complete pairs (131 DZ and 164 MZ) and 4 incomplete pairs (2 MZ and 2 DZ).

### 2.2 Questionnaires

The Vineland Adaptive Behaviour Scale II (VABS‐II; Sparrow et al. 2005) is a questionnaire used to measure adaptive behaviour. It has been validated across different conditions with problems in communication, social and motor difficulties (Balboni et al. 2001). VABS comprises four domains: communication, socialization, daily living skills and motor skills. Communication includes subdomains of receptive, expressive, and written language skills. In infancy this domain includes reactions to sounds and voices and expressing needs and pleasure through sounds and gestures. Socialization measures behaviour related to interpersonal relationships, play and leisure time, as well as coping skills. In infancy, socialization comprises behaviours related to responding to caregivers’ behaviour and showing interests, as well as showing emotions through noises, gestures, or expressions. Daily living skills include personal, domestic and community related skills, while the motor skills subscale is comprised of gross and fine motor skills. Motor skills in infancy are related to, for example, head support, sitting, and reaching/grabbing behaviour. Each item in the domains is scored from 0 (never) to 2 (usually), depicting how often the behaviour is observed. All subscales can be measured in infants under 1 year of age. However, as daily living skills only have three questions that are applicable for under‐year‐old children, with most questions relating to independent self‐care, household chores and community related skills, it was not used in the analysis. Standardised scores are provided for each domain (mean = 100, standard deviation = 15) and general score for adaptive (adaptive behaviour composited, ABC, mean = 100, standard deviation = 15). Raw scores were used as preliminary analysis suggested the need to combine socialization and communication into one domain (social‐communication). Covariates residualised from motor and social‐communication were age, term age and household income. The residualised raw scores were modelled in the analysis to investigate the aetiological structure of early adaptive behaviour.

### 2.3 Statistical Analyses

The analysis plan was pre‐registered in Open Science Framework (OSF; https://osf.io/fp4q9/) after data collection and preliminary analysis. Based on visual inspection of the score distribution, skewness, and number of values, social and communication skills were combined into one domain (see Figure S1). As the number of items in the socialization and communication domains for infants under 1 year of age are limited but cover similar behaviours, for example, cries when hungry or wet, makes sounds of pleasure, and makes sounds/gestures to get attention (communication domain); and shows one or two emotions, and makes/tries to make social contact through noise/smiles/and so forth (socialization domain), this combination was deemed to be appropriate. Next, we tested for possible significant associations between VABS scales and potential covariates, that is, age, term age, sex, parental age, education, and family income. The robust sandwich estimator in generalized estimating equations (GEE) was used to account for correlations between twin pairs (Carlin et al. 2005). The GEE analysis found that age and gestation age had positive associations with both motor skills and social‐communication domains (Table 2). Models were adjusted for these associations by modelling residualised scores.

**TABLE 2:** The GEE analysis with VABS‐II domains at 5 months of age and demographic data.

	Beta	95% CI	*p*
**Motor**			
Age (in days)	0.28	0.19, 0.37	**< 0.001**
Sex	0.1	−0.12, 0.32	0.38
Term age	0.17	0.02, 0.21	**0.02**
Parental age	−0.07	−0.19, 0.05	0.23
Parental education	−0.08	−0.189, 0.04	0.177
Income	−0.19	−0.30, −0.07	**< 0.001**
**Social‐communication**			
Age (in days)	0.15	0.03, 0.27	**0.02**
Sex	0.21	−0.01, 0.43	0.06
Term age	0.11	0.00, 0.22	**0.05**
Parental age	−0.04	−0.16, 0.07	0.48
Parental education	−0.06	−0.17, 0.05	0.30
Income	−0.11	−0.23, 0.02	0.09

Abbreviations: GEE, generalized estimating equations; VABS‐II, Vineland Adaptive Behaviour Scale.

Bolding indicates *p* < 0.05.

Twin studies use a genetically informed method that breaks down the individual variability in a trait into genetic and environmental factors. First, monozygotic and dizygotic twin correlations (i.e., the correlation between twin 1 and twin 2) were calculated for each trait. An MZ correlation higher than the DZ correlation suggests genetic influence. Secondly, a saturated model (that models the observed data) was fitted, as well as submodels to test the assumptions of twin modelling (equal means and variances across twins and zygosity, compared to the saturated model; see Table S1 in Supplementary Material). Next, multivariate modelling was used to investigate the heritability of the traits defined by VABS scales of social‐communication skills and motor skills and decompose covariance between traits into genetic and environmental factors. Based on the cross‐twin cross‐trait correlations (see Table 3), we selected an ACE model to decompose covariance among scales into additive genetics (*A*), shared environment (*C*; everything that makes twins similar to each other that is not due to genetics), and unique environment factors (*E*; everything that makes twins different from each other, including measurement error). Given the bivariate nature of our multivariate problem, the correlated factor solution was selected for modelling purposes. In this model, an ACE model is fitted to each measure, then estimates are computed for the degree to which *A*, *C*, and *E* correlate between the different traits. Model fit was compared on log‐likelihood and Bayesian Information Criterion Schwarz 1978; however, model solution was selected based on BIC value only, as a more conservative index for model identification. Data analysis was performed in R 4.1.2 (R Core Team 2021), and twin model fitting was performed through maximum likelihood optimization with the R package OpenMx, version 2.19.8 (Neale et al. 2016). For assessment of statistical power see Supplementary Material S1.

**TABLE 3:** Twin–twin, phenotypic, and cross‐twin cross‐trait correlations split between monozygotic and dizygotic twins with 95% confidence intervals.

	Phenotypical	MZ	DZ
**Twin**–**twin**			
Motor		0.88 (0.84, 0.91)	0.77 (0.70; 0.83)
Social‐communication		0.90 (0.87; 0.92)	0.84 (0.79; 0.88)
**Cross‐twin cross‐trait**	0.30 (0.20; 0.39)	0.26 (0.16; 0.36)	0.29 (0.19; 0.39)

### 2.4 Genome‐Wide Polygenic Scores

Genotyping of DNA samples was done using Infinium Global Screening Array (Illumina). Standard procedures were used for processing and quality control; see Falck‐Ytter et al. (2021) for a description, and Supplementary Material S2 for additional details. The use of polygenic scores requires a discovery dataset and a target dataset. The discovery dataset is used to identify SNPs (single nucleotide polymorphisms) that are associated with the trait. Results from the discovery dataset are then used to derive polygenic scores in the target dataset by creating a weighted sum of all the alleles that either increase or decrease the chance of the outcome of interest. As discovery datasets for this study, we used the results of GWAS's of autism (*n* cases = 18,381, *n* controls = 27,969; Grove et al. 2019), ADHD (*n* cases = 20,183, *n* controls = 35,191; Demontis et al. 2019), bipolar disorder (*n* cases = 20,352, *n* controls = 31,358; Stahl et al. 2019), major depressive disorder (*n* cases = 246,363, *n* controls = 561,190; Howard et al. 2019), and schizophrenia (*n* cases = 69,369, *n* controls = 236.642; Ripke et al. 2020). Our current sample of 5‐month‐old twins was used as the target dataset. The polygenic scores were calculated using the PRS‐CS (polygenic prediction via Bayesian regression and continuous shrinkage priors) method (Ge et al. 2019), which is described elsewhere (Falck‐Ytter et al. 2021), but see Supplementary Material S2 for additional details. To strive for inclusivity, all available data were used in the analyses and compared to the more common approach based on restriction to European ancestry. The associations between VABS scores and polygenic scores were calculated using the robust sandwich estimator in GEE separately for each polygenic score and were uncorrected for multiple comparisons. The first 10 principal components of ancestry were included as covariates.

## 3 Results

MZ twins are genetically identical at conception, while DZ twins only have half of their genetic makeup in common. Thus, MZ twins having higher twin correlations compared to DZ indicate genetic influence, while similar twin correlations suggest shared environmental factors. The results for twin correlations for both VABS domains suggest that both genetic and shared environmental components are significant in adaptive behaviour (Table 3). All twin model assumptions were met (see SM), and a univariate ACE model was used, as it did not provide a significantly worse fit compared to the saturated model. The ACE model comparison confirmed that additive genetics, shared and unique environments all contributed to the early emergence of adaptive behaviour (Table 4). While phenotypic correlation was significant, cross‐twin cross‐trait correlations suggested no additive genetic influences on the covariance between social‐communication and motor skills (Table 3).

**TABLE 4:** Variance decomposition into genetic and environmental components according to the correlated factors solution for the bivariate model of adaptive functioning.

	*A*	*C*	*E*	rA	rC	rE	biv_A	biv_C	biv_E
**Motor**	0.21	0.67	0.12	−0.39 [−1; 0.08]	0.45 [0.29; 0.61]	0.32 [0.17; 0.45]	−0.06 (−0.21)	0.33 (1.09)	0.04 (0.12)
	[0.10; 0.36]	[0.52; 0.77]	[0.09; 0.16]						
**Social‐communication**	0.12	0.78	0.10						
	[0.04; 0.22]	[0.68; 0.86]	[0.08; 0.13]						

*Note*: Estimates of aetiological factors and correlations are reported with 95% confidence intervals shown in brackets, while the proportion of phenotypic correlation explained is reported for bivariate estimates of shared aetiological influences.

Abbreviations: *A*, % variance explained by additive genetics (equivalent to univariate estimates); biv_A, shared variance explained by additive genetics (bivariate heritability); biv_C, shared variance explained by shared environment; biv_E, shared variance explained by unique environment; *C*, % variance explained by shared environment (equivalent to univariate estimates); *E*, % variance explained by unique environment (equivalent to univariate estimates); rA, genetic correlation; rC, correlation of shared environment; rE, correlation of unique environment.

**FIGURE 1:**
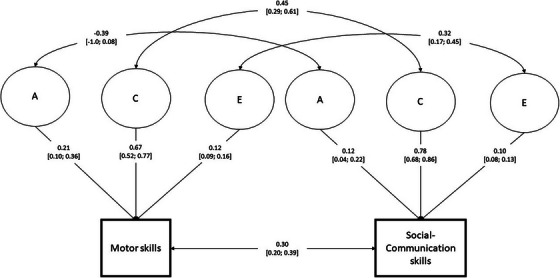
Correlated factors model solution to the bivariate model of adaptive behaviour measures. Observed measures are represented by squares, and latent factors by circles. Variance partitions and aetiological correlations are reported with 95% confidence intervals.

A comparison of bivariate twin models found that a correlated factors solution did not significantly differ from the fully saturated model and was used as the bivariate twin model (see Table 5). The estimates of the ACE model (Figure 1) suggested that motor skills were weakly heritable (*A* = 0.21, 95% CI: 0.10, 0.36), while there was a large influence by shared environment (*C* = 0.67, 95% CI: 0.52, 0.77). The rest of the variance was explained by contributions of unique environment (*E* = 0.12, 95% CI: 0.09, 0.16). Social‐communication skills also had weak, but significant heritability (*A* = 0.12, 95% CI: 0.04, 0.22), but similarly to motor skills, the largest contribution came from shared environment (*C* = 0.78, 95% CI: 0.68, 0.86), with unique environment accounting for the rest (*E* = 0.10, 95% CI: 0.08, 0.13). There was no significant genetic overlap between motor and social‐communication skills (*r* = −0.37, 95% CI: −1.0; 0.08). Meanwhile, there was a moderate overlap of influences from both shared environments (*r* = 0.45, 95% CI: 0.29, 0.61) and unique environments (*r* = 0.32, 95% CI: 0.17, 0.45) between social‐communication and motor skills, with shared and unique environments explaining 0.33 and 0.04 of the shared variances, respectively.

**TABLE 5:** Model fit statistics for the correlated factors model solution (in bold) compared to the fully saturated model.

Model	# Parameters	−2LL	df	AIC	ΔLL	Δdf	*p*
**Fully saturated**	28	2329.98	1119	2385.98	—	—	
**Correlated factors**	**11**	**2346.79**	**1136**	**2368.79**	**16.81**	**17**	**0.47**

Abbreviations: −2LL, log‐likelihood fit statistics; AIC, Akaike information criterion; df, degrees of freedom; Δdf, difference in degrees of freedom from the reference model; ΔLL, difference in log‐likelihood fit statistics from the reference model.

We tested associations between motor and social‐communication skills and polygenic scores for autism, ADHD, schizophrenia, depression, and bipolar disorder (Table 6). No statistically significant associations were found between polygenic scores and any of the adaptive behaviour domains. The results remained similar when restricting the analyses to participants with purely European ancestry (see Tables S2–S3).

**TABLE 6:** Associations between polygenic scores and VABS scores (motor skills and social‐communication skills, respectively), calculated using GEE analyses.

	Motor skills			Social‐communication skills		
	*β* (95% CI)	Std error	*p*	*β* (95% CI)	Std error	*p*
**Autism**	0.05 (−0.06; 0.17)	0.06	0.38	0.16 (<−0.01; 0.23)	0.06	0.06
**ADHD** ^a^	0.11 (<−0.01; 0.22)	0.06	0.05	0.06 (−0.04; 0.16)	0.05	0.21
**Schizophrenia**	0.02 (−0.11; 0.16)	0.07	0.75	0.08 (−0.04; 0.19)	0.06	0.20
**Bipolar disorder**	−0.01 (−0.17; 0.14)	0.08	0.88	−0.03 (−0.18; 0.12)	0.08	0.69
**MDD** ^b^	0.05 (−0.06; 0.16)	0.06	0.39	0.01 (−0.09; 0.11)	0.05	0.88

*Note*: The first 10 principal components of ancestry were included as covariates.

Abbreviations: GEE, generalized estimating equations; VABS, Vineland Adaptive Behaviour Scale.

^a^Attention deficit hyperactivity disorder.

^b^Major depressive disorder.

## 4 Discussion

In this study we analysed the genetic and environmental influences on adaptive behaviour in early infancy, finding that shared environment explained most of the variance in adaptive behaviour at 5 months of age. In early infancy caregivers play an integral role in a child's development, as the child is highly dependent on them and the opportunities for development that they present at home. This could be related to toys and play, exposure to language or chances provided to explore the environment.

VABS‐II is a parental questionnaire, meaning that parents report behaviour they observe. If the same person rates both twins, it is possible that this reflects on shared environmental influence and higher correlations between twins (Saudino 2017). Future work should formally investigate the potential bias introduced by parent ratings assessing the same measures from different informants (Neale and Cardon 2013). However, it is unlikely that our findings would be explained solely by rater bias. In that case, we would expect the bias to affect the different scores uniformly and induce large overlap across the different estimates of shared environmental influences. While we found significant correlations between shared environment influences on social‐communication and motor skills (rC = 0.45), the residual variance indicates independent influences from shared environment acting uniquely on the different domains, which cannot be fully explained by parent ratings. Caregiver reports during infancy offer invaluable data on an infant's daily behaviour that might not be observed in a strange environment with unfamiliar adults. While Flom et al. (2018) suggested that caregivers might have difficulties separating twin behaviour, leading to an inflated shared environment factor, and that the differences might be easier to differentiate over time, an argument can be made that in early infancy caregivers might know their children the best.

The result that social‐communication skills were mostly explained by shared environment was in line with previous literature, as language development in 2‐year‐olds suggests high shared environmental influence (i.e., Dale et al. 2000). Additionally, our confidence intervals for heritability estimates overlap those of pooled confidence interval estimates of both language and basic interpersonal functions presented by Austerberry et al. (2022) meta‐analysis. However, our confidence intervals for shared environment overlapped with language, but not basic interpersonal functions. This could be due to our combined measure of social‐communication encompassing both of these measures. Marrus et al. (2015) suggest that due to the role of caregivers in early childhood, shared environment is more important for social behaviour earlier in life than later. During the first year of life, infants are spending almost all their time with their caregivers, and they develop social skills through interactions with them. Caregiver's speech has been found to affect children's language development in early childhood (e.g., Huttenlocher et al. 2010). In addition, attachment patterns that are important for children's social development and formed at an early age have been shown to be highly influenced by shared environment (Bokhorst et al. 2003). However, later in development children can choose their environment to a higher extent, leading to gene‐environment correlation exacerbating the influence of genes later in development. Cheung et al. (2015) suggest that as children gain more control over their own environment, they strengthen their genetic advantages/disadvantages and have the potential to simultaneously promote multiple abilities through these experiences, increasing the role of genes later in life.

Our finding of the importance of shared environment in the development of social‐communication skills supports this theory and shows that shared environment is especially important in infancy. This offers possibilities for supporting the development of these skills that might help meet the demands of the environment later. Caregivers’ behaviour and home environment in infancy are vital in early social development, and support provided at home could offer a way to nurture social development, especially for children that might have initial vulnerabilities.

Previous studies of motor skills have reported primarily genetic and non‐shared environmental influences (e.g., Saudino 2012), in contrast to our findings of low genetic effect and high shared environmental effect. While the confidence intervals for our heritability estimates overlap with that of Austerberry et al.’s psychomotor function pooled estimates, the confidence interval for shared environment did not. Some additional caution should be warranted for potential caregiver rating bias, as a study by Bussu et al. (2023) found that at 5 months of age genes and non‐shared environments explained the variability of motor development, when cognitive assessment was done by a researcher. However, there have also been some studies that have found significant shared environmental influences, when looking at motor milestones. A study by Peter et al. (2012) found that shared environment explained most early motor milestones, while Goin‐Kochel et al. (2008) found that in their sample of twins with autism, VABS‐II scores, including motor skills, were partially explained by genetic factors and had some influence of shared environment. Cheung et al. (2015) found that while the genetic influence on gross motor skills was relatively unchanged across time points, fine motor was found to have low heritability and high shared environment influence in early childhood. As our measure for adaptive behaviour is comprised of both gross and fine motor skills instead of separating them, this could explain some of our shared environment findings. It could also be that the effect of shared environment is important in very early infancy but becomes less relevant approaching toddlerhood. It is possible that after children reach certain motor milestones, like walking, they are less dependent on their caregivers for environmental stimuli and have more opportunities to use their motor skills.

In this study, the shared environmental factors for social‐communication and motor skills correlated with each other, and 33% of the shared environment was shared between them at 5 months of age. Previous studies have found that higher motor skills lead to higher levels of cognitive functioning (Oudgenoeg‐Paz et al. 2015; Veldman et al. 2019). In a study by Oudgenoeg‐Paz et al. (2015), attainment of sitting unsupported and the age of walking were associated with spatial memory and later spatial language. However, as there were no hierarchical relations in our model, our results suggest that in early infancy there are some environmental factors that promote both sets of these skills. This could possibly mean caregiver factors, such as what type of play they engage in with the infant, and what kind of toys and stimuli children are provided during early infancy that might support the development of both skills. While we discuss shared environment in relation to caregivers and home environment, it is important to note that shared environment refers to all factors that make twins more similar to each other (regardless of zygosity). This means that shared environmental influence might stem from pre‐ and postnatal exposures not related to the caregiving routines of the parents. It is also important to note that unique environmental influence does not imply that parenting is unimportant. It has been shown that parents of older children treat their children somewhat differently (e.g., Meunier et al. 2012), meaning that some of the parental influence can manifest as unique environmental influences.

There was no significant shared genetic effect between the adaptive behaviour domains. While not significant, the negative direction between genetic influences on social‐communication and motor skills was surprising, as it suggests the directions of the genetic influences are opposite to each other. This would mean that there are some genetic factors that could increase the variance of motor function while simultaneously decreasing the variance of social‐communication skills or vice versa. Larger twin studies are needed to elucidate the potential associations between motor and social‐communication skills at this age.

The associations between adaptive behaviour and polygenic scores for neurodevelopmental or psychiatric conditions did not reach statistical significance. This could be explained by polygenic scores currently lacking predictive power to detect these associations (Plomin and von Stumm 2022). In addition, while adaptive behaviour related phenotypes have been associated with polygenic scores of neurodevelopmental and psychiatric conditions in previous studies, these associations have been found in older infants (e.g., Askeland et al. 2022; Verhoef et al. 2024), and thus associations might emerge later in development.

There are some limitations to this study. Due to the small sample size, issues with power are present, and twin analyses should be considered exploratory. Post hoc power analysis was performed to examine the reliability of our findings. While our study had enough power to estimate environmental and unique environmental effects and correlations, our analyses were underpowered for additive genetic effects and correlation. Thus, caution should be given when interpreting the results regarding genetics, and twin studies with a larger sample size are needed to test the significance of genetic correlations further. In addition, the scale in VABS‐II items is between 0 and 2, and there are a limited number of items that are applicable at 5 months of age. Including other methods outside of caregiver reports might provide more comprehensive and objective picture of heritability and environmental factors regarding adaptive behaviour.

Additionally, our sample is Sweden based with high socioeconomic status that could limit the generalisability of these findings, as high socioeconomic status has been shown to associate with gene‐environment effects (e.g., Finkel et al. 2022; Kirkpatrick et al. 2015; Tucker‐Drob et al. 2011; Turkheimer and Horn 2014). High socioeconomic status has been found to associate with higher heritability estimates, while low socioeconomic status is associated with lower heritability and higher shared environmental estimates in relation to cognitive/mental abilities (Kirkpatrick et al. 2015; Tucker‐Drob et al. 2011; Turkheimer and Horn 2014). However, in a longitudinal infant study by Tucker‐Drob et al. (2011), heritability accounted for small variation in mental ability at 10 months of age regardless of socioeconomic status, but by 2 years of age those with higher socioeconomic status had higher genetic estimates, while those of lower socioeconomic status had less change of heritability and environmental estimates. Thus, while our sample is of high socioeconomic status, differences at the age of 5 months might not yet be as prominent as later in life. Yet, the majority of replications regarding associations between socioeconomic status and shared environmental effects have been found in American studies that have higher variation in participant ethnicities compared to European studies and those of more homogenous samples (Tucker‐Drob et al. 2011; Turkheimer and Horn 2014), suggesting a possible wider societal/cultural effect to that of estimates of heritability and environment outside of socioeconomic status. Additionally, it should be noted that adaptive behaviour as a construct should be seen as a culturally specific construct that describes the capabilities to meet societal expectations (Tassé et al. 2012). For example, children might be raised more communally, thus having a different caregiver environment than in cultures with a more individualistic environment. There might be differences in toilet training practices and thus also expectations relating to that. Hence, cultural bias should be considered when deciding on adaptive behaviour measures and making comparisons, as the aim of adaptive behaviour is to reflect an individual's performance in relation to the expectations of their surrounding culture.

Even with these limitations, this is one of a few twin studies looking at a parent rated adaptive behaviour in a community sample at 5 months of age, providing deeper understanding of the aetiology of adaptive behaviour in early infancy. While, according to the standard definition, adaptive behaviour reflects individuals’ typical behaviour, not their best performance, this distinction is difficult to do in early infancy. Hence, it is interesting to conduct longitudinal studies of the current sample to establish long‐term correlates (phenotypic and etiological) both in terms of later adaptive function and later cognition.

In conclusion, shared environment seems to play an important part in shaping individual differences in infant adaptive behaviour, a result with potential real‐life applications. It is important to find a way to provide opportunities and support to develop the skills needed to meet the expectations of everyday life, as this could help people that have difficulties or vulnerabilities regarding adaptive behaviour. As multiple neurodevelopmental conditions have been shown to be associated with difficulties in adaptive behaviour (e.g., Perry et al. 2002), understanding different ways that shared environment contributes to the development of adaptive behaviour in infancy could provide better tailored support for individuals with difficulties in adaptive behaviour and families with a history of neurodevelopmental conditions.

## Ethics Statement

This study was approved by the regional ethics board in Stockholm and was conducted in accordance with the Declaration of Helsinki.

## Conflicts of Interest

Tony Charman has served as a paid consultant to F. Hoffmann‐La Roche Ltd. and Servier; and has received royalties from Sage Publications and Guilford Publications.

## Supporting information




**Figure S1**: Score distribution of Vineland‐II for A) Socialization, B) Communication, and C) combined social‐communication domains.
**Figure S2**: Post‐hoc power analysis for bivariate model for motor skills (trait 1) and social‐communication (trait 2). *A = additive genetic effects; C = shared environmental effects; E = unique environment effects; cor = correlations*.
**Figure S3**: Principal component analysis based on the BATSS sample and HapMap Phase III, and SweGen reference data. Values over 0.062 indicate Swedish/European ancestry (n = 508). 82 participants had values between 0.01 and 0.062, and 4 participants had values under .0.01.
**Table S1**: Analysis of assumption for univariate twin models of motor and social‐communication skills. The fully saturated model is the baseline that models the means and variances across zygosity for each twin in a pair. Model 1 compares means across twins within a pair; Model 2 compares means across zygosity; Model 3 compares variances across twins within a pair; Model 4 compares variances across zygosity.
**Table S2**: Polygenic Scores, when 86 participants with values under 0.062 for the principal component of European ancestry were removed (n = 508).
**Table S3**: Polygenic Scores, when 4 participants with values under 0.01 for the principal component of European ancestry were removed (n = 590).

## Data Availability

The data that support the findings of this study are available from the corresponding author upon reasonable request. Note that sharing of pseudonymized personal data will require a data processor agreement (DPA), according to Swedish and EU law. Data‐analysis scripts are publicly available at https://github.com/brainhabit/BT‐VABS https://anonymous.4open.science/r/BT‐VABS‐A480/. The data are not publicly available due to privacy or ethical restrictions.
