# Supplementary material for: Genetic and Environmental Effects on Parent‐Rated Adaptive Behaviour in Infancy
Source: Dev Sci. 2025 Jun 19;28(4):e70041. doi: 10.1111/desc.70041 (PMC12179425; doi:10.1111/desc.70041)
Supplement: Supplementary file 1 — Figure S1: Score distribution of Vineland‐II for A) Socialization, B) Communication, and C) combined social‐communication domains. Figure S2: Post‐hoc power analysis for bivariate model for motor skills (trait 1) and social‐communication (trait 2). A = additive genetic effects; C = shared environmental effects; E = unique environment effects; cor = correlations. Figure S3: Principal component analysis based on the BATSS sample and HapMap Phase III, and SweGen reference data. Values over 0.062 indicate Swedish/European ancestry (n = 508). 82 participants had values between 0.01 and 0.062, and 4 participants had values under .0.01. Table S1: Analysis of assumption for univariate twin models of motor and social‐communication skills. The fully saturated model is the baseline that models the means and variances across zygosity for each twin in a pair. Model 1 compares means across twins within a pair; Model 2 compares means across zygosity; Model 3 compares variances across twins within a pair; Model 4 compares variances across zygosity. Table S2: Polygenic Scores, when 86 participants with values under 0.062 for the principal component of European ancestry were removed (n = 508). Table S3: Polygenic Scores, when 4 participants with values under 0.01 for the principal component of European ancestry were removed (n = 590). [file DESC-28-e70041-s001.docx]

Supplementary Material

**Genetic and environmental effects on parent-rated adaptive behaviour in infancy**

Visual inspection of score distribution

**A**
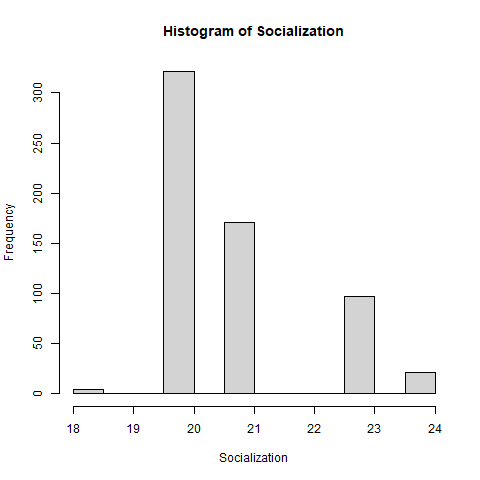
**B**
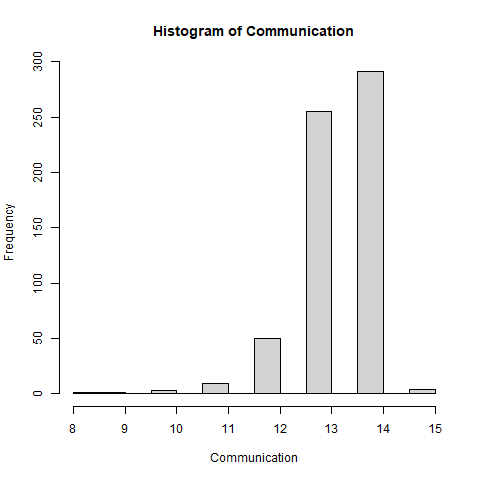


**C**
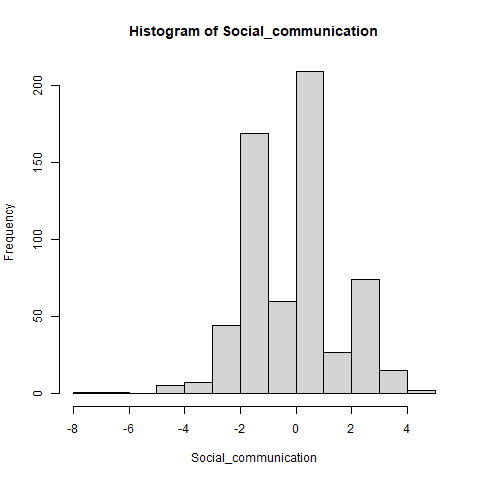


**Figure S1.** Score distribution of Vineland-II for A) Socialization, B) Communication, and C) combined social-communication domains.

Supplementary material S1.

*Assessment of statistical power.* The results described in the current paper were part of a larger project (BATSS) that consists of a vast battery of experiments and assessments. Prior to data collection, a general, broad scope power analysis was conducted. In this we assumed that 225 pairs of twins would have valid data, and a ratio of MZ/DZ to be .5. A study of this size approaches 97% power to detect a heritability of 40%, and shared environmental effect of 40% for a phenotype. With the assumption that two variables would have 40% heritability, 40% of variation would be due shared environment, and that the phenotypic correlation would be r = .40, and to the same degree be mediated by genetic and shared environmental effects, we would reach a nearly 88% power to detect a significant genetic contribution to a correlation between these two measures.

Post-hoc power analysis was performed to assess reliability of our findings. Specifically, Monte Carlo simulations were used to estimate the statistical power of detecting significant genetic and environmental influences in a bivariate twin model framework based on the etiological estimates from the correlated factors solution model reported in the previous sections. (See [https://anonymous.4open.science/r/BT-VABS-C01F](https://github.com/brainhabit/BT-VABS)  for script.)

Simulations (n = 1046) were used to generate data according to a variance/covariance matrix based on the estimated genetic and environmental parameters obtained from our bivariate model with the addition of random variations. Simulated datasets were generated for each twin pair and reflecting the zygosity distribution observed in our sample (n=299 twin pairs; n=166 monozygotic).
For each simulation, the same correlated factor solution model was fitted and the statistical significance of parameter estimates was evaluated by comparing the full model with nested sub-models through Likelihood Ratio Tests (LRT). Reduced models were built to test the significance of specific variance components by constraining the corresponding parameters to zero. This was performed separately for additive genetic effects on the 2 traits (i.e., A1 and A2); shared environmental effects (i.e., C1 and C2); unique environment effects (i.e., E1 and E2); and correlations (i.e., rA, rC, rE).

Significance of each variance component and correlation was determined at the α = 0.05 level. If the likelihood ratio test indicated a significant drop in model fit upon fixing a parameter to zero, the parameter was deemed significant for that simulation. Power was estimated as the number of significant estimates for each parameter divided by the number of simulations performed.

Results are shown in Figure S2.

We highlight that results showed that our study had enough power (≥80%) to estimate environmental effects, both from shared (pC1=pC2=0.89) and unique environment (pE1=pE2=0.89), and their correlations (p_rC=0.86; p_rE=0.80). However, our analyses were underpowered for additive genetic effects (pA1=0.51; pA2=0.04) and genetic correlation (p_rA=0.11).

***
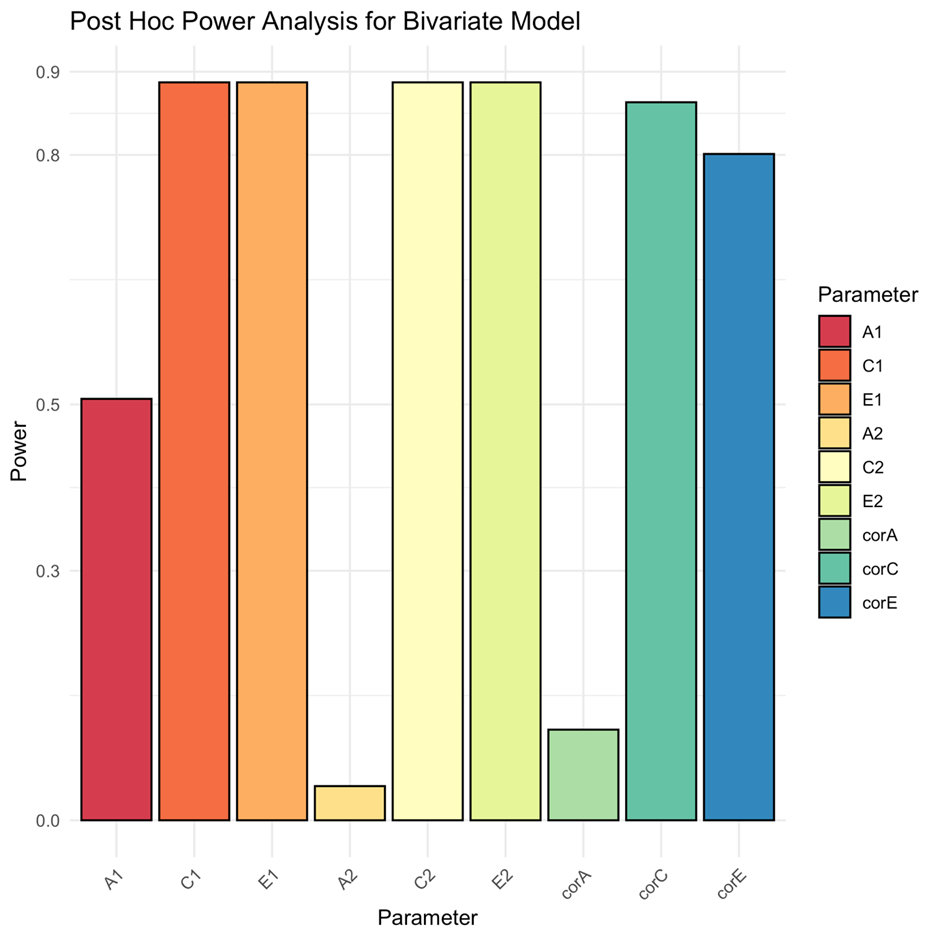
***

**Figure S2.** Post-hoc power analysis for bivariate model for motor skills (trait 1) and social-communication (trait 2). *A = additive genetic effects; C = shared environmental effects; E = unique environment effects; cor = correlations.*

| **Motor Skills** |  |  |  |  |  |  |  |
| --- | --- | --- | --- | --- | --- | --- | --- |
| **Model** | **# Parameters** | **-2LL'** | **df** | **AIC** | **ΔΧ²** | **Δdf** | **p** |
| Fully Saturated Model | 10 | 1202.027 | 563 | 1222.027 | NA | NA | NA |
| Model 1 | 8 | 1202.145 | 565 | 1218.145 | 0.118 | 2 | 0.943 |
| Model 2 | 7 | 1204.272 | 566 | 1218.272 | 2.245 | 3 | 0.523 |
| Model 3 | 5 | 1205.539 | 568 | 1215.539 | 3.512 | 5 | 0.622 |
| Model 4 | 4 | 1205.562 | 569 | 1213.562 | 3.535 | 6 | 0.739 |
|  |  |  |  |  |  |  |  |
| **Social-Communication** |  |  |  |  |  |  |  |
| **Model** | **# Parameters** | **-2LL'** | **df** | **AIC** | **ΔΧ²** | **Δdf** | **p** |
| Fully Saturated Model | 10 | 1177.930 | 564 | 1197.930 | NA | NA | NA |
| Model 1 | 8 | 1179.139 | 566 | 1195.139 | 1.210 | 2 | 0.546 |
| Model 2 | 7 | 1179.203 | 567 | 1193.203 | 1.273 | 3 | 0.735 |
| Model 3 | 5 | 1182.586 | 569 | 1192.586 | 4.657 | 5 | 0.459 |
| Model 4 | 4 | 1188.390 | 570 | 1196.390 | 10.460 | 6 | 0.107 |

**Table S1.** Analysis of assumption for univariate twin models of motor and social-communication skills. The fully saturated model is the baseline that models the means and variances across zygosity for each twin in a pair. Model 1 compares means across twins within a pair; Model 2 compares means across zygosity; Model 3 compares variances across twins within a pair; Model 4 compares variances across zygosity.

*Abbreviations: -2LL = log-likelihood fit statistics; df = degrees of freedom; BIC = Bayesian Information Criterion;* Δ*LL = difference in log-likelihood fit statistics from the reference model;* Δ*df = difference in degrees of freedom from the reference model*

Supplementary material S2.

*Quality control and Imputation*. The array consists of 730 059 markers of which 97.8% had a sample call rate 98% with average SNP call rate per sample 99.3%. We performed both quality control (QC) for the raw genotyping and imputed calls at the individual and marker level using PLINK v1.90. Both autosomal and X chromosomes were imputed using IMPUTE2. For imputation, we used the European reference panel from 1000 genomes phase III. For calculation of the polygenic scores using PRS-CS, we first changed the format of each summary statistics file to match the format requirement according to instructions from the PRS-CS, followed by calculating the posterior SNP effect size estimates using the imputed genotype file, summary statistics, and LD reference data from 1000 Genome phase III. We used the parameters recommended by Ge et al. (2019) of which all others were the default exempt the phi (PARAM_PHI), which was fixed to 1e-2 as also recommended.

The following QC criteria were used to identify the outlier individuals and remove the low-quality markers: individual QC: discordant sex, heterozygosity rate > 3SD, individual genotype failure rate > 0.03, and relatedness; marker QC: info score < 0.8, minor allele frequency < 0.01, Hardy-Weinberg equilibrium < 1e-06, individual missingness < 0.1, and marker missingness < 0.05. There were 518 570 markers remained after genotyping QC and 7 507 876 markers after imputation QC.

*Genetic ancestry.* The base rate of specific SNPs can vary across populations, and genetic ancestry can therefore affect the validity of polygenic score analyses. To control for this, we need to quantify genetic ancestry. Therefore, a principal component analysis (PCA byEIGENSOFT 7.2.1) was performed based on the BATSS sample and two resources of reference data — HapMap Phase III (HapMap3), representing individuals with genetic ancestry from Asia, Africa, and Europe, and SweGen, representing the genetic ancestry of the Swedish population (Ameur et al., 2017; Gibbs et al., 2003) —to analyse population stratification of our twin sample in comparison to the reference datasets (using R3.6.3). (See Figure S3.) Visual inspection of the results of the PCA confirmed that the majority of the sample had Swedish/European ancestry (n=508). When we restricted our analyses to participants with purely European ancestry, the results remained similar. See Table S2 and S3.


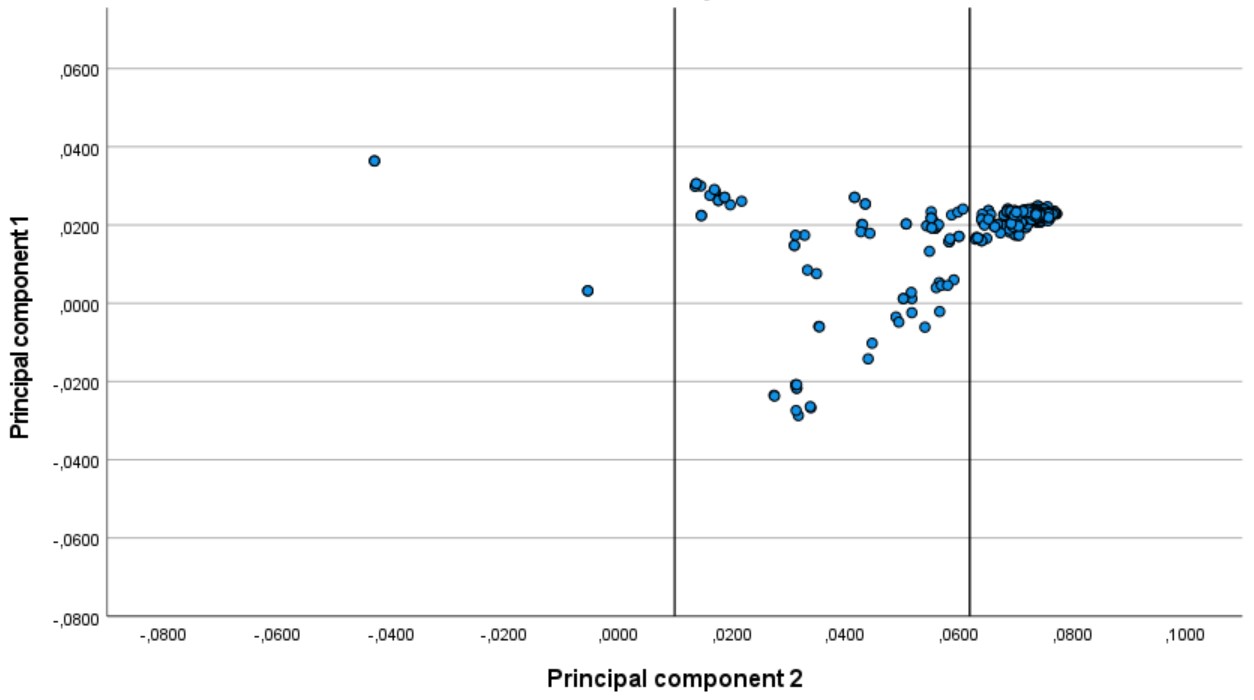


**Figure S3.** Principal component analysis based on the BATSS sample and HapMap Phase III, and SweGen reference data. Values over 0.062 indicate Swedish/European ancestry (n=508). 82 participants had values between 0.01 and 0.062, and 4 participants had values under .0.01.

**Table S2.** Polygenic Scores, when 86 participants with values under 0.062 for the principal component of European ancestry were removed (n=508):

|  | ***Motor skills*** | | | ***Social-communication skills*** | | |
| --- | --- | --- | --- | --- | --- | --- |
|  | ***β (95% CI)*** | ***Std error*** | ***p*** | ***β (95% CI)*** | ***Std error*** | ***p*** |
| ***Autism*** | *0.05 (-0.08; 0.18)* | *0.07* | *0.45* | *0.06 (-0.06; 0.18)* | *0.06* | *0.32* |
| ***ADHD^a^*** | *0.09 (-0.03; 0.20)* | *0.06* | *0.14* | *0.04 (-0.06; 0.14)* | *0.05* | *0.45* |
| ***Schizophrenia*** | *-0.02 (-0.17; 0.13)* | *0.08* | *0.76* | *0.06 (-0.06; 0.19)* | *0.07* | *0.33* |
| ***Bipolar disorder*** | *0.01 (-0.17; 0.19)* | *0.09* | *0.91* | *-0.02 (-0.19; 0.15)* | *0.09* | *0.79* |
| ***MDD^b^*** | *0.06 (-0.05; 0.18)* | *0.06* | *0.28* | *-0.003 (-0.11; 0.10)* | *0.05* | *0.95* |

**Table S3.** Polygenic Scores, when 4 participants with values under 0.01 for the principal component of European ancestry were removed (n=590):

|  | ***Motor skills*** | | | ***Social-communication skills*** | | |
| --- | --- | --- | --- | --- | --- | --- |
|  | ***β (95% CI)*** | ***Std error*** | ***p*** | ***β (95% CI)*** | ***Std error*** | ***p*** |
| ***Autism*** | *0.06 (-0.06; 0.18)* | *0.06* | *0.32* | *0.12 (<-0.01; 0.23)* | *0.06* | *0.05* |
| ***ADHD^a^*** | *0.11 (<-0.01; 0.22)* | *0.06* | *0.05* | *0.06 (-0.04; 0.16)* | *0.05* | *0.22* |
| ***Schizophrenia*** | *0.02 (-0.12; 0.16)* | *0.07* | *0.83* | *0.08 (-0.04; 0.19)* | *0.06* | *0.19* |
| ***Bipolar disorder*** | *-0.01 (-0.17; 0.14)* | *0.08* | *0.85* | *-0.03 (-0.18; 0.12)* | *0.08* | *0.68* |
| ***MDD^b^*** | *0.05 (-0.06; 0.16)* | *0.06* | *0.41* | *0.01 (-0.09; 0.11)* | *0.05* | *0.88* |

References

Ameur, A., Dahlberg, J., Olason, P., Vezzi, F., Karlsson, R., Martin, M.,Viklund, J., Kähäri, A. K., Lundin, P., Che, H., Thutkawkorapin, J.,Eisfeldt, J., Lampa, S., Dahlberg, M., Hagberg, J., Jareborg, N.,Liljedahl, U., Jonasson, I., Johansson, Å., … Gyllensten, U. (2017).SweGen: A whole-genome data resource of genetic variability in a cross-section of the Swedish population. *European Journal of Human Genetics, 25*,1253–1260.

Ge, T., Chen, C.-Y., Ni, Y., Feng, Y.-C. A., & Smoller, J. W. (2019). Polygenic prediction via Bayesian regression and continuous shrinkage priors. Nature Communications, 10(1), 1776. https://doi.org/10.1038/s41467-019-09718-5

Gibbs, R. A., Belmont, J. W., Hardenbol, P., Willis, T. D., Yu, F., Yang, H.,Ch’ang, L. Y., Huang, W., Liu, B., Shen, Y., Tam, P. K. H., Tsui, L. C.,Waye, M. M. Y., Wong, J. T. F., Zeng, C. Q., Zhang, Q. R., Chee, M.S., Galver, L. M., Kruglyak, S., … Tanaka, T. (2003). *The International Hapmap Project.* Deep Blue, University of Michigan.
